# Comparison of deep learning segmentation and multigrader-annotated mandibular canals of multicenter CBCT scans

**DOI:** 10.1038/s41598-022-20605-w

**Published:** 2022-11-03

**Authors:** Jorma Järnstedt, Jaakko Sahlsten, Joel Jaskari, Kimmo Kaski, Helena Mehtonen, Ziyuan Lin, Ari Hietanen, Osku Sundqvist, Vesa Varjonen, Vesa Mattila, Sangsom Prapayasotok, Sakarat Nalampang

**Affiliations:** 1 Medical Imaging Centre, Department of Radiology, Tampere University Hospital, Teiskontie 35, 33520 Tampere, Finland; 2 Aalto University School of Science, Maarintie 8, 02150 Aalto, Finland; 3 Planmeca Oy, Asentajankatu 6, 00880 Helsinki, Finland; 4 Division of Oral and Maxillofacial Radiology, Faculty of Dentistry, Chiang Mai University, Suthep Rd., T. Suthep, A. Muang, Chiang Mai, Thailand; 5 Alan Turing Institute, British Library, 96 Euston Rd, London, NW1 2DB UK; 6 Department of Computer Science, Aalto University School of Science, Maarintie 8, 02150 Espoo, Finland

**Keywords:** Cone-beam computed tomography, Machine learning, Medical imaging

## Abstract

Deep learning approach has been demonstrated to automatically segment the bilateral mandibular canals from CBCT scans, yet systematic studies of its clinical and technical validation are scarce. To validate the mandibular canal localization accuracy of a deep learning system (DLS) we trained it with 982 CBCT scans and evaluated using 150 scans of five scanners from clinical workflow patients of European and Southeast Asian Institutes, annotated by four radiologists. The interobserver variability was compared to the variability between the DLS and the radiologists. In addition, the generalisation of DLS to CBCT scans from scanners not used in the training data was examined to evaluate its out-of-distribution performance. The DLS had a statistically significant difference (*p* < 0.001) with lower variability to the radiologists with 0.74 mm than the interobserver variability of 0.77 mm and generalised to new devices with 0.63 mm, 0.67 mm and 0.87 mm (*p* < 0.001). For the radiologists’ consensus segmentation, used as a *gold standard*, the DLS showed a symmetric mean curve distance of 0.39 mm, which was statistically significantly different (*p* < 0.001) compared to those of the individual radiologists with values of 0.62 mm, 0.55 mm, 0.47 mm, and 0.42 mm. These results show promise towards integration of DLS into clinical workflow to reduce time-consuming and labour-intensive manual tasks in implantology.

## Introduction

There has recently been a rapid increase of studies demonstrating that Artificial Intelligence methodologies, especially those based on deep learning neural networks, can distinguish structural patterns in medical imaging data with excellent accuracy^1^, and serve radiologists as augmenting tools for clinical workflow^2^. However, in dental and maxillofacial radiology, so far there have been relatively few studies that have used deep learning approaches for localising or segmenting the bilateral mandibular canals^3–8^**,** each hosting a neurovascular bundle containing an artery, veins, and the inferior alveolar nerve. The mandibular canals have two openings; the foramen mandibulae posterior in the ramus area and foramen mentale anterior in the parasympheal area. What makes the canal localization in CBCT images challenging is that there are a number of anatomical variations in the pathway and shape of the canal, and also ethnic variability is known to play a role^9,10^. As the inferior alveolar nerves supply motor and sensory innervations, any damage to them can cause temporary or permanent nerve injuries. In order to avoid compression or other surgical complications, in implantology, a 2 mm safety margin above the mandibular canal is recommended^11^. Therefore, the accurate knowledge of mandibular canal locations is extremely important for various oral and maxillofacial surgical operations, and in the diagnosis of neurogenic, vascular, or adjacent lesions to the canals.

The first studies using deep learning convolutional neural network (CNN) approach to automatically localise the bilateral mandibular canals in CBCT images appeared as recently as in 2020^3,4^. In the first study, multiple CNNs were used on images of 102 patients from a single scanner, where it was found that a 3D U-Net CNN could outperform 2D CNNs with a mean intersection over union value of 0.577^3^. At the same time, another study^4^ also proposed a 3D U-Net style CNN approach, but using a larger number of clinically heterogeneous CBCT images of 637 patients using four different scanners. As the performance measures, they reported the average symmetric surface distance value of 0.45 mm, mean curve distance value of 0.56 mm, and Dice similarity coefficient (DSC) value of 0.61.

Recently two other studies have used CNNs for mandibular canal segmentation with multiple CBCT scanners using a smaller number of images^5,6^. First of these studies^5^ used 235 patient images from four scanners and the second study^6^ used 138 patient images from three scanners, and they reported DSC values of 0.774 and 0.580, respectively. There have also been two studies that have used a rather small number of images from a single CBCT scanner^7,8^. The first of these studies^7^ used 187 patient images and reported the DSC value of 0.57, mean intersection over union value of 0.70, and mean curve distance value of 0.62 mm as the performance measures. The second study^8^ used 81 multiplanar reconstructed patient images and reported the DSC value of 0.93, average symmetric surface distance value of 0.16 mm, and mean curve distance value of 1.59 mm as the performance measures.

Although the previous works have shown promising results, the majority of these studies have had datasets with limited clinical diversity in terms of ethnicity and patient age groups, and there have been only limited or no description on patient specific heterogeneities. In addition, the algorithms have not been compared to the assessments of multiple radiologists to account for the interobserver variability, and the generalisation of these systems to CBCT images from new scanners has not been comprehensively studied. In these works, there are a variety of generally used and valid performance measures such as the Dice similarity coefficient, the mean intersection over union, the average symmetric surface distance, and the mean curve distance. The first three of these measures are used to evaluate the segmentation performance and the mean curve distance measures the deviation between the paths of two curves, namely between the ground truth and the prediction curves^4,7,8^.

In the present study, we focus on validating a fully convolutional neural network-based deep learning system (DLS) for mandibular canal segmentation, introduced in a recent study^4^, from the clinical and technical points of view. To comprehensively analyse the system, we use a larger and clinically more heterogeneous dataset of CBCT scans than reported in any of the previous studies. Indeed, our database has images from five different CBCT scanners from four different vendors and patient cohorts of two different ethnicities that we use to train a new version of the DLS.

In the clinical validation process, we compare the performance of the DLS against four experienced dental and maxillofacial radiologists. Performance is estimated by comparing the interobserver variability between the radiologists and evaluate if there is a statistically significant difference to the variability between the deep learning system and the radiologists, with the null hypothesis of “the deep learning system and radiologists do not differ in the segmentation performance”. For technical validation, we analyse the out-of-distribution generalisation capability of a version of the deep learning system^4^, which was trained with 509 images from ProMax 3D Max/Mid and Scanora 3Dx devices. This includes temporal and geographical generalisation, as we analyse three different devices with a portion of the imaged patients from another country as well as more recent images from previously used devices. The generalisation capability is similarly evaluated by observing if there is a statistically significant difference to the interobserver variability, with the null hypothesis of “the deep learning system and radiologists do not differ in the segmentation performance for scans from new CBCT devices”. To evaluate the quantitative performance between the DLS and the radiologists, we construct consensus segmentations from the four radiologists’ segmentations, and evaluate it and the radiologists on them. Moreover, to analyse the qualitative performance, a senior radiologist evaluated the automatic and expert segmentations to identify the types of errors in them. The contributions of this study are novel performance evaluation of a deep learning neural network approach when compared to the interobserver variability of multiple radiologists and out-of-distribution generalisation capability in terms of new scanners and ethnicity in dental and maxillofacial radiology CBCT imaging.

## Methods

### Data collection and cohort description

The CBCT imaging data was acquired from the Cranio and Dentomaxillofacial Radiological Department of the University Hospital of Tampere (TAUH), Finland as the first cohort, and from the Department of Oral Radiology, Faculty of Dentistry, Chiang Mai University (CMU), Thailand as the second cohort. All the data in this study is from a normal clinical workflow that represents pre- and postoperative examinations of patients of 10 to 95 years old. The reasons for radiological examination include normal findings and anatomy, but also various traumas, benign or malign pathological conditions, and syndromes. The CBCT scans were randomly and retrospectively selected and pseudonymised, before the annotation process.

The collected dataset of CBCT scans consisted of 1103 individuals, with 869 Finnish patients (79%) and 234 Thai patients (21%). In the Finnish population, with the mean age of 53.7 years, 56% were females and 44% males. The Thai patient population with the mean age of 39.8 years, consisted of 51% females and 49% males. In the dataset, 649 CBCT scans (57%) were imaged using ProMax 3D Max/Mid; (Planmeca, Helsinki, Finland), 125 (11%) using Viso G7; (Planmeca, Helsinki, Finland), 124 (11%) using Scanora 3Dx; (Soredex, Tuusula, Finland), 120 (11%) using DentiScan; (The National Science and Technology Development Agency, Pathum Thani, Thailand), and 114 (10%) using GiANO HR; (NewTom, Bologna, Italy). The first three scanners were used in the first cohort and the latter two scanners in the second cohort. The scan resolution ranged from 0.1 to 0.6 mm isotropic voxel-sizes with most commonly 0.2 mm (59%), 0.4 mm (23%) or 0.3 mm (13%). All the volumes were resampled into 0.4 mm isotropic voxel spacing using linear interpolation before deep learning analysis. Human annotators had access to the scans in the original resolution and could augment the view of the scans using software tools.

**Figure 1:**
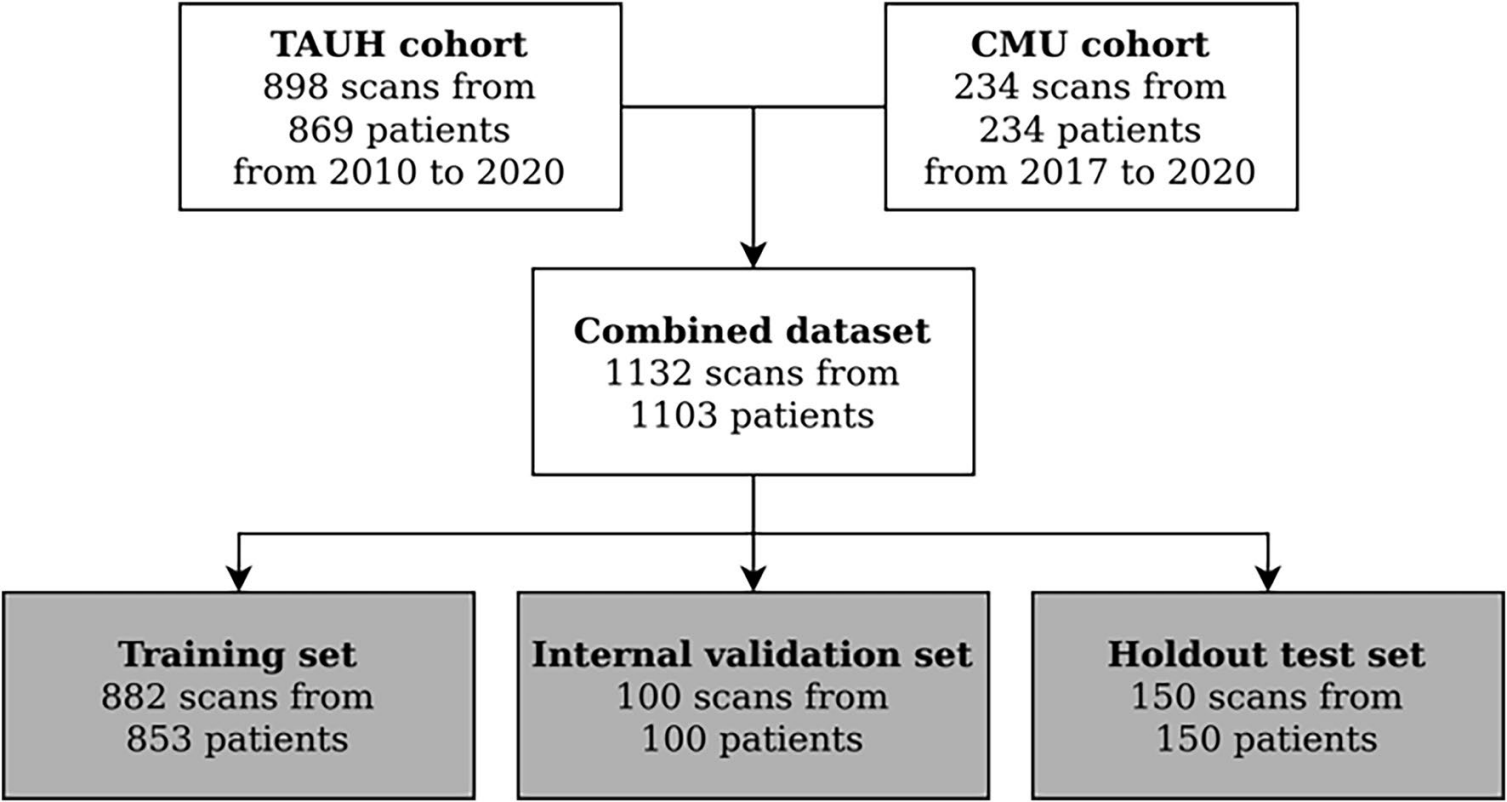
Flowchart for data collection. Patients were selected from recent clinical workflows at the Cranio and Dentomaxillofacial Radiological Department of the University Hospital of Tampere, Finland (TAUH), and from the Department of Oral Radiology, Faculty of Dentistry, Chiang Mai University (CMU), Thailand. Combined dataset had a total number of 1132 scans from 1103 patients that were split into a training set, internal validation set, and holdout test set with 853, 100, and 150 patients, respectively.

The deep learning methodology is driven by data such that in order to estimate the performance of the model, a partition of the data is held out and only used for validation of the results. This set is called the test set and it was randomly selected with uniform distribution of scanners. The rest of the data was used for the DLS development, and thus called the development set. Flowchart for data collection is presented in Fig. 1.

The mandibular canal was annotated using Planmeca developed Romexis 4.6.2. software, which has a built-in tool for mandibular canal annotation using control points and spline interpolation. The control points were standardised to be 3 mm apart from each other, and in the foramen mentale curvature area the canal was annotated using multiple control points, when necessary. Four dentomaxillofacial radiologists participated in this study referred to as Expert 1–4. Experts 1, 3, and 4 are working as senior specialists in TAUH, having 11–35 years of experience in dentistry and Expert 2 who works as a senior consultant in the private sector with 35 years of experience in dentistry. The annotation of the development set was performed by Experts 3 and 4 with 90% and 10% proportions of the set, respectively. The test set was annotated independently by all radiologists. Since the end-point of the mandibular canal in the mandibular foramen region is ambiguous, the end-point was selected to match the shortest annotation in the superior direction, thus improving the sensitivity of the canal localization in the foramen mentale and dental regions rather than highlighting differences in the canal path lengths. The set was also subjectively annotated for the clarity of mandibular canal visibility to be either Clear or Unclear. The test set scans were also annotated for the following conditions if present: movement artefact, bisagittal osteotomy, metal artefact, difficult pathology, and difficult bone structure including difficult anatomy and osteoporosis.

This study is based on a retrospective and registration dataset and as such does not involve experiments on humans and/or the use of human tissue samples and no patients were imaged for this study. A registration and retrospective study does not need ethical permission or informed consent from subjects according to the law of Finland (Medical Research Act (488/1999) and Act on Secondary Use of Health and Social Data (552/2019)) and according to European General Data Protection Regulation (GDPR) rules 216/679. The use of the Finnish imaging data was accepted by the Tampere University Hospital Research Director, Finland October 1, 2019 (vote number R20558). Certificate of Ethical Clearance for the Thai imaging data was given by the Human Experimentation Committee, Faculty of Dentistry, Chiang Mai University, Thailand (vote number 33/2021) July 5, 2021. According to the Thailand legislation informed consent was not needed.

### Validation of the deep learning system

**Figure 2:**
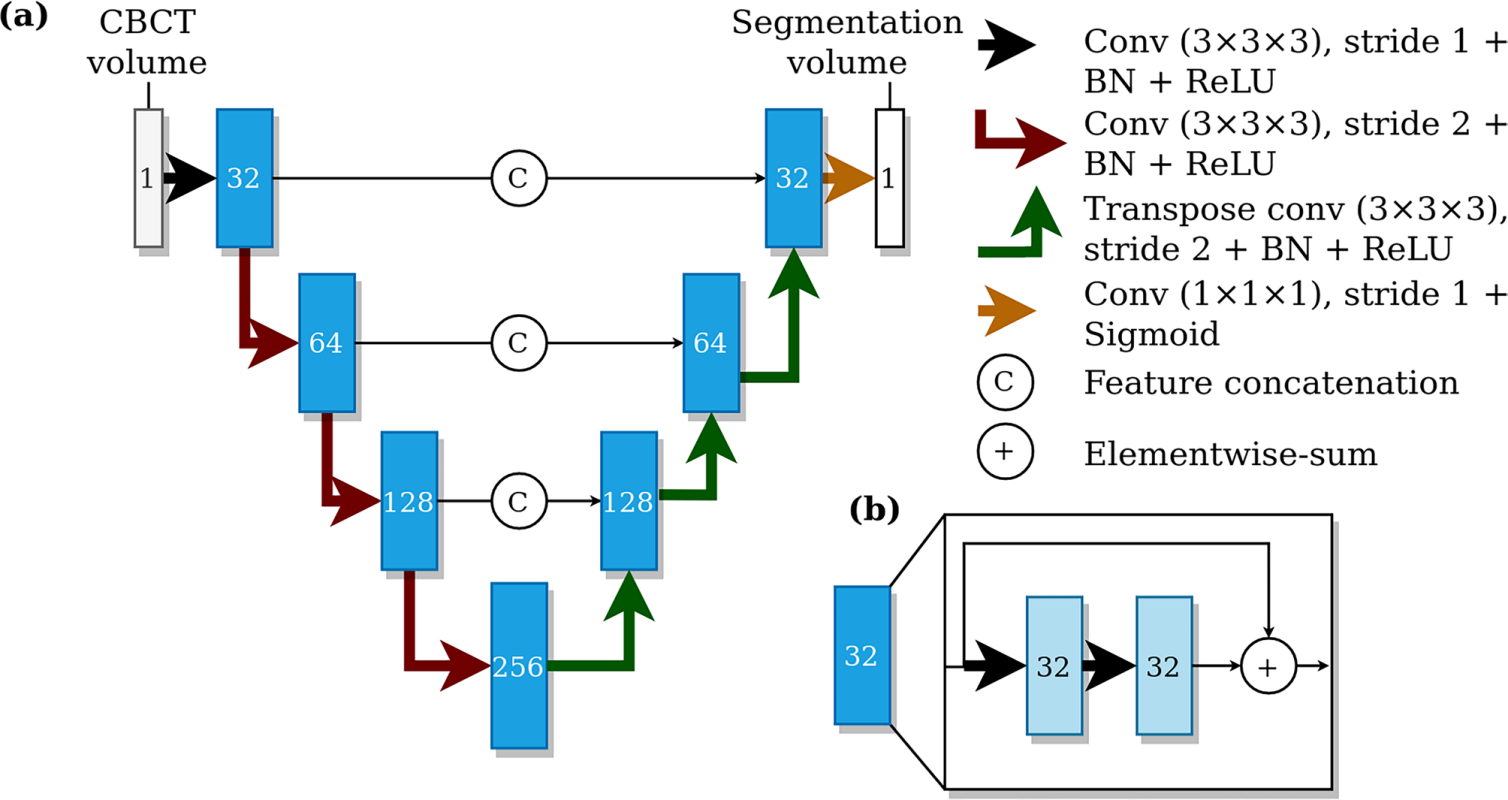
The architecture of the deep learning system. (**a**) A U-net style deep learning system architecture with a contracting pathway and an expanding pathway. The contracting pathway is visualised with red arrows, each marking a convolution block with a stride of two. The expanding pathway is visualised with green arrows, each marking a transpose convolution block with a stride of two. The pathways are connected with feature concatenation marked using arrows with the letter C. The number of feature maps is shown on each block and all the convolutions except the last one have a kernel size of 3 × 3 × 3. The last convolution has one feature, a kernel size of 1 × 1 × 1, no layer normalisation, and uses the sigmoid nonlinearity. (**b**) Each block includes two convolutions with a stride of one, batch normalisation (BN), and ReLU non-linearities with elementwise summation marked using arrows with a plus sign.

We utilised the previously proposed CNN-based method^4^ as our deep learning model, i.e. a type of fully convolutional neural network using a U-net style architecture^12^. The model utilises three dimensional convolutional layers that enables the recognition of patterns in axial, coronal, and sagittal planes, simultaneously. The model was trained with volume patches from randomly flipped and translated volumes using the Dice-loss objective for 60 epochs. The model parameters were updated using the Adam optimizer^13^ and the final parameters were selected from checkpoints after each training epoch, based on the best validation set performance. The model architecture is shown in Fig. 2. We developed an improvement to the canal extraction post processing algorithm from the model segmentation. In short, mandibular canal route segments are obtained from the CNN output using a skeletonization routine^14^, route segments are concatenated using a heuristic, and then the routes with anatomical characteristics of mandibular canals are selected. Finally, the pair of routes with the most symmetricity is chosen as the pair of canals.

### Statistical analysis

For the evaluation of the mandibular canal localization performance, we used the mean curve distance (MCD), similar to previous works^4,7,8^, and in addition we propose the symmetric mean curve distance (SMCD). In the case of MCD, for each point, i.e. a three dimensional coordinate, on a *ground truth* curve, the distance to the closest point on another *estimator* curve is computed and then these distances are averaged. Thus, it estimates the average distance from one curve to another in three dimensional space. However, by definition, the MCD is computed from the point of view of the *ground truth* curve, effectively measuring the *sensitivity* in curve localization, and thus, there are cases when the MCD does not reflect the errors well. For example, if the *ground truth* curve and *estimator* curves are well aligned, but the *estimator* curve is longer from either or from both ends. Hence, we propose the SMCD measure, which is calculated as the average of the MCD values computed both ways. In addition, the SMCD is useful in summarising the interobserver variability, as the role of the *ground truth* and *estimator* curves are not well defined. Note that a visualisation of how the MCD is computed is presented in the Supplementary.

In mathematical terms, let *T* and *E* be the sets of points that define the *ground truth* curve and the *estimator* curve, respectively. We perform the mandibular canal segmentation in three dimensional space, and thus the points are the three dimensional coordinates of the discretized mandibular canal path curves. The *point to curve distance* function *d(x,S)* is defined such that it computes the minimum Euclidean distance from a point *x* to the set of points *S* that defines a curve:

1 $$d\left(x,S\right)=\underset{s\in S}{min}\parallel x-s \parallel_{2}.$$

Then the MCD is computed as:

2 $$MCD\left(T,E\right)=\frac{1}{|T|}\sum_{t\in T}d\left(t,E\right).$$

The SMCD is computed using Eq. (2) and a permutation of the arguments:

3 $$SMCD\left(T,E\right)=\frac{1}{2}\left(MCD\left(T,E\right)+MCD\left(E,T\right)\right).$$

We have also evaluated the proportion of the canal path within a 2 mm radius in order to evaluate the localization accuracy that approximates the 2 mm safety margin above the canal that is used as a guideline for implant planning^11^. Volumetric segmentation measures, such as the Dice similarity coefficient, were deemed unsuitable for our main results, since the annotation tool in our study was designed to use a fixed diameter for the canal. However, the results measured with the Dice similarity coefficient are reported in the Supplementary Figs. S2–S5.

We evaluated and analysed the variability between the Experts and the deep learning system by comparing the radiologists’ canal annotations and the segmentations produced by the deep learning system in a pairwise manner. In order to estimate the highest level of interobserver variability, for each CBCT scan we selected the pair of Expert annotations with the highest mean curve distance. Similarly, we selected one Expert annotation with the highest mean curve distance with the deep learning system, by treating the Expert annotation as the *ground truth curve* and the automatic segmentation as the *estimator*, to estimate the highest variability between the system and an Expert. The generalisation capacity of the previously published system^4^ was evaluated similarly by selecting the Expert annotation with the highest mean curve distance to the segmentation produced by the system.

We estimated the objective performance between the Experts and the deep learning model by constructing a label voting scheme of the expert annotations as the reference ‘ground truth’ using SimpleITK library^15^ and evaluated the performance using SMCD. Specifically, voxels are assigned a background or mandibular canal label with maximum votes and undecided voxels are marked as a canal label. After this, the segmentation was skeletonized and the curve was determined with connected component analysis. Statistical significance of all the main results were computed using the two-tailed Wilcoxon signed-rank test with alpha value selected at 0.001 using *statannotations* Python package^16^.

### Inclusion/exclusion criteria

There was no exclusion criteria in the diagnostically acceptable patient scans.

## Results

### Patient cohorts

The dataset used in this study includes 982 and 150 CBCT scans from 953 and 150 patients for the development and test set, respectively. All reported results are computed for the test set, in which each scan included the two mandibular canals. The sample characteristics are presented in Table 1 while the distribution of the devices of the subsets are shown in Table 2 for the development set and holdout test set. The deep learning system training and internal validation sets are described in detail in Table S1.

**Table 1:** Characteristics of the study sample with percentages in parentheses.

Parameter	Cohort 1 (n = 869)	Cohort 2 (n = 234)
Age (y)^a^	54.9 ± 33.2	39.8 ± 17.0
**Gender**		
Female	487 (56)	119 (51)
Male	382 (44)	115 (49)
**Race**		
Caucasian	869 (100)	2 (1)
Asian	0 (0)	232 (99)

Cohort 1 from University Hospital of Tampere (TAUH) and Cohort 2 from Chiang Mai University (CMU).

^a^Mean and standard deviation.

**Table 2:** Distribution of scans with five CBCT scanners and patients for each of the subsets.

Manufacturer and device	Development		Holdout	
	Scans	Patients	Scans	Patients
Planmeca ProMax (3D, 3D Max, 3D Mid)	619 (63%)	590 (62%)	30 (20%)	30 (20%)
Planmeca Viso G7	95 (10%)	95 (10%)	30 (20%)	30 (20%)
Soredex Scanora 3Dx	94 (10%)	94 (10%)	30 (20%)	30 (20%)
NSTDA DentiScan	90 (9%)	90 (9%)	30 (20%)	30 (20%)
NewTom GiANO HR	84 (9%)	84 (9%)	30 (20%)	30 (20%)
Total	982	953	150	150

Unambiguous heterogeneities were marked with at least one expert label: movement artefact (7, 2%), metal artefact (9, 3%), and bisagittal osteotomy (4, 1%). More ambiguous heterogeneities were assessed by using majority voting: difficult bone structure including difficult anatomy and osteoporosis (99, 33%), and difficult pathology (3, 1%). There were 275 (92%) canals marked Clear and 25 (8%) Unclear, also determined using majority voting. Comparison of the performance in each heterogeneity group is reported in Supplementary Fig. S1.

### Interobserver variability

**Figure 3:**
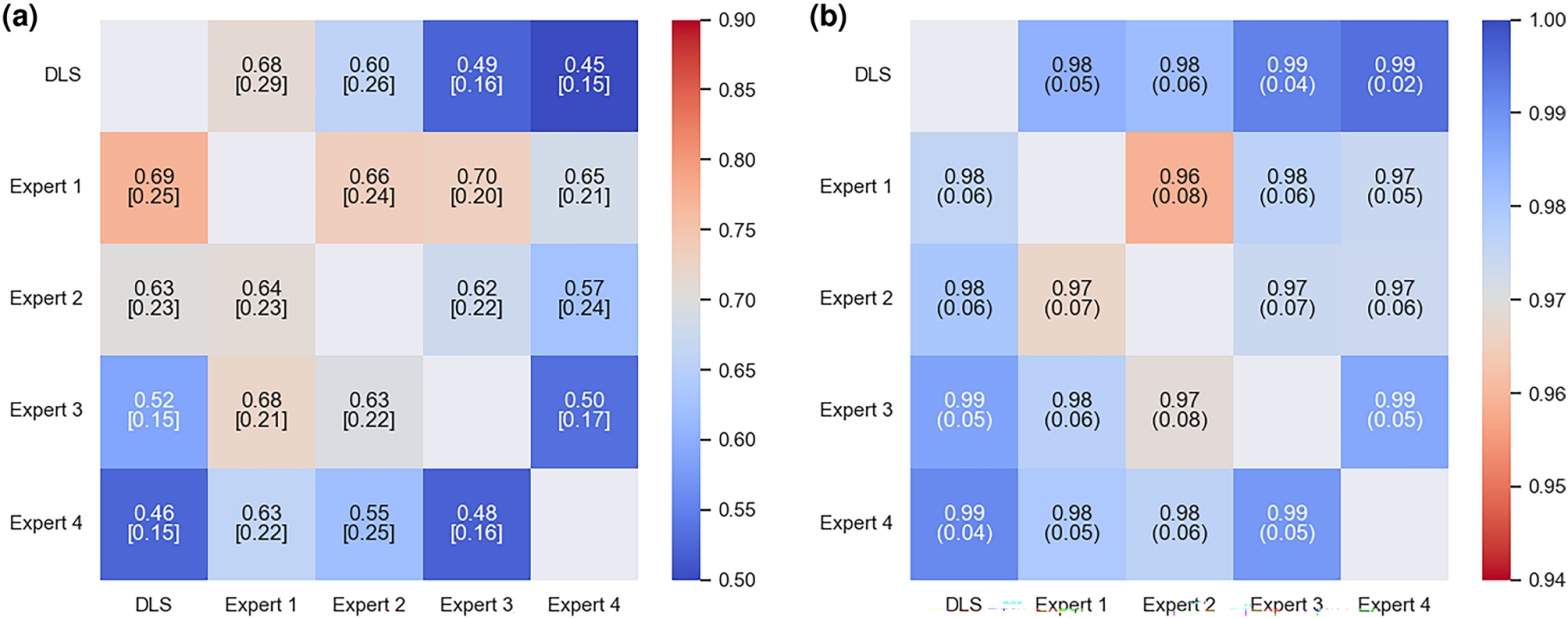
Pairwise comparison of the Experts and the deep learning system (DLS). Each row represents which assessment was used as the ground truth and each column which was used as the estimate. The mean curve distance measure is asymmetric, which results in asymmetric matrices. (**a**) Median and interquartile range [IQR] of curve distances in millimetres. (**b**) The mean (SD) of the proportion of the canal within a 2.0 mm margin.

The results of the individual variability analysis are presented in Fig. 3, where the Experts and the deep learning system are compared in a pairwise manner for all the possible combinations, to obtain a fine-grained analysis. In Fig. 3a, the MCD results are presented. The DLS and Expert 4 had overall the highest agreement measure with 0.46 mm median MCD and the lowest agreement with Expert 1 with 0.69 mm median MCD. In terms of the interobserver variability, the highest agreement was with Expert 3 and Expert 4 with 0.48 mm median MCD and the lowest agreement between Expert 1 and Expert 3 with 0.70 mm median MCD. The proportion of the canal within the specified 2 mm margin, as evaluated in a pairwise manner, is presented in Fig. 3b. On average, all the observers had very similar performance, indicating that most of the time the annotated canal paths did not vary more than 2 mm, as the largest difference of 96%, which was between Expert 1 and Expert 2.

**Figure 4:**
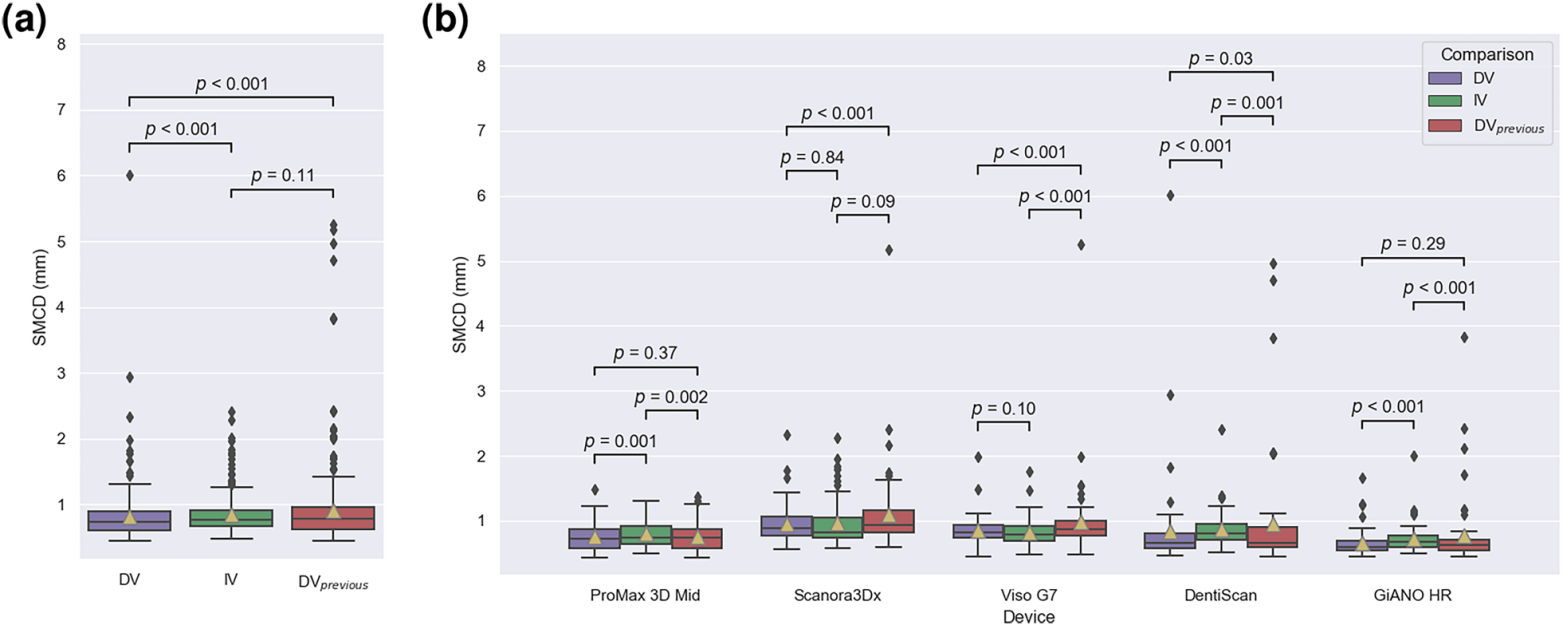
Tukey’s boxplot comparison of interobserver variability (IV), DLS to expert variability (DV), and previous method^4^ to expert variability (DV_previous_), measured in symmetric mean curve distance (mm). Statistical significance measured with Wilcoxon signed-rank test. (**a**) Comparison of full test dataset (N = 300). (**b**) Device-wise comparison between the groups (N = 60 per device).

In the evaluation of the highest variability, there was a statistically significant difference in the mean rank of highest interobserver and model variability (*p* < 0.001) with the DLS having lower median SMCD. The median [interquartile range] and mean (standard deviation) of the highest Expert to Expert, i.e. interobserver, SMCD were 0.77 [0.25] mm and 0.84 (0.28) mm, respectively, whereas the highest DLS to Expert SMCD median [interquartile range] was 0.74 [0.28] mm and mean (standard deviation) 0.81 (0.41) mm. In the device-wise comparison, the DLS had a non-significant difference with highest variability performance against the Experts on Scanora3Dx (*p* = 0.84) with 0.89 [0.29] mm and Viso G7 (*p* = 0.10) with 0.82 [0.19] mm, a significant difference with lower median SMCD on DentiScan (*p* < 0.001) with 0.66 [0.22] mm, GiANO HR (*p* < 0.001) with 0.60 [0.15] mm, and ProMax 3D Mid (*p* < 0.001) with 0.72 [0.29] mm. The full results are presented in Fig. 4 and a scatter plot comparison is presented in Supplementary Fig. S4.

The previously published system was able to produce results for 299 out of 300 canals with the highest variability analysis having a median of SMCD of 0.78 [0.33] mm and mean (standard deviation) 0.91 (0.63) mm, with a significant difference to the current system (*p* < 0.001). In the device-wise comparison, the previously reported system had a non-significant difference with highest variability performance against the Experts on Scanora3Dx (*p* = 0.09) with 0.93 [0.34] mm, a significant difference with lower median SMCD on DentiScan (*p* = 0.001) with 0.67 [0.31] mm and GiANO HR (*p* < 0.001) with 0.63 [0.17] mm, and a significant difference with a higher median SMCD on ProMax 3D Mid (p = 0.002) with 0.74 [0.34] mm and Viso G7 (*p* < 0.001) with 0.87 [0.21] mm. The full results are presented in Fig. 4.

### Reference segmentation

**Figure 5:**
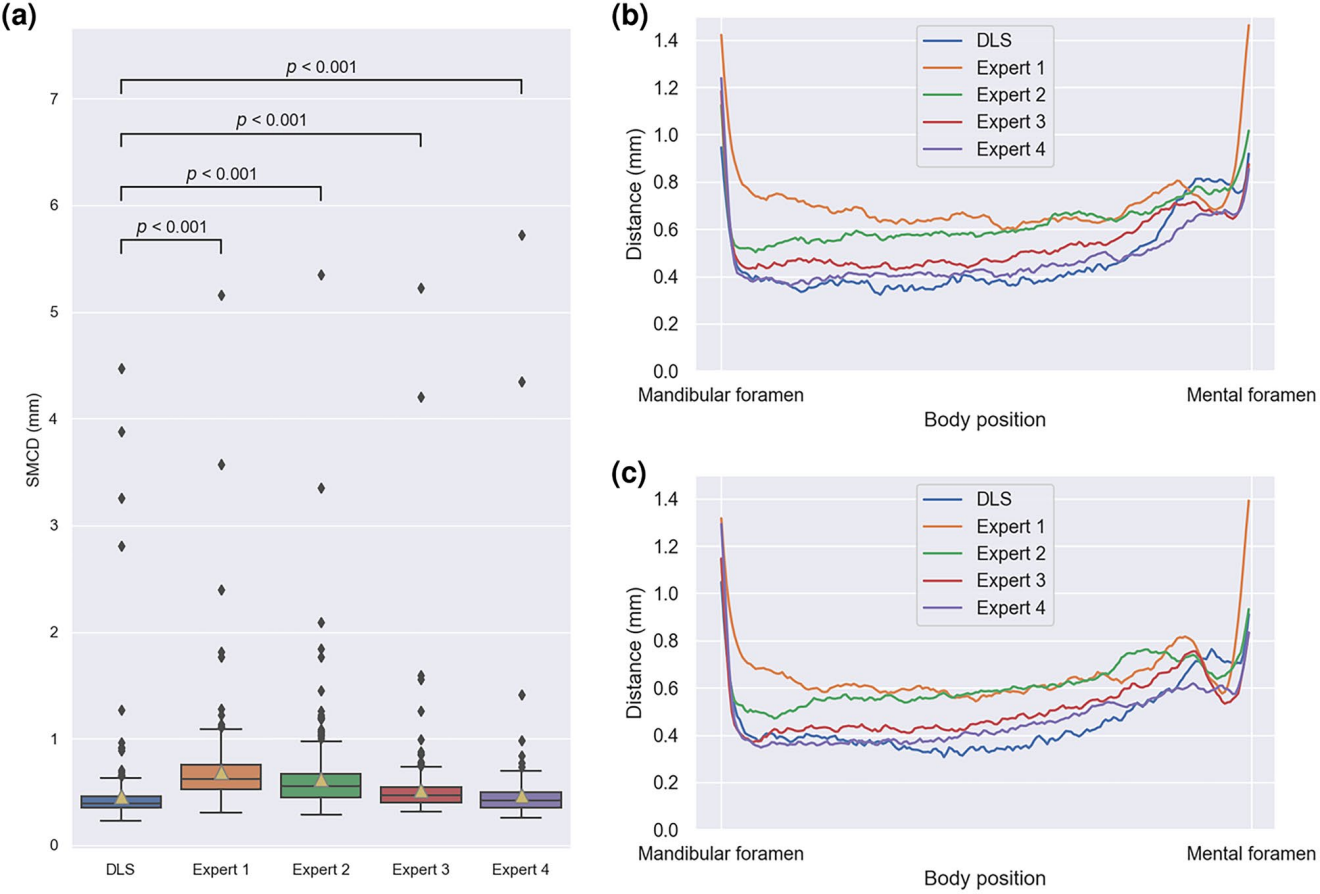
Measuring interobserver variability and DLS performance against a majority voted reference segmentation from expert assessments. (**a**) Tukey’s boxplot comparison of the performances of DLS and the experts against the reference segmentation. (**b**) SMCD in millimetres with respect to the approximate position of the left canals (**c**) SMCD with respect to the approximate position of the right canals.

There was a statistically significant difference in the performance between all the Experts and the deep learning system against the reference segmentation (*p* < 0.001). Expert 1 had the largest median [interquartile range] of SMCD with 0.62 [0.23], Expert 2 with 0.55 [0.22], Expert 3 with 0.47 [0.14], and Expert 4 the lowest with 0.42 [0.14] mm, whereas the DLS had the smallest median SMCD of 0.39 [0.11] mm. In addition, the DLS had the lowest mean (standard deviation) of SMCD with 0.46 (0.39) mm, while Expert 1 had mean of SMCD of 0.68 (0.38), Expert 2 0.62 (0.39), Expert 3 0.52 (0.38), and Expert 4 0.47 (0.40) mm. There were considerable missing parts in the reference segmentation for a total of two canals, as there was no agreement between the radiologists’ segmentations. These are seen as the two outliers with highest SMCD results for all the Experts and amongst the outliers of the DLS. The results are presented in Fig. 5a and Table S2.

We also examined, for both the left and right canals, how the distance to the reference annotation is related to on which approximated position of the mandibular canal curve it is measured. In practice, 200 uniformly spaced interpolation points were used to have a dense representation of the curve, and the point to curve distance was computed using Eq. (1). A visual examination of Fig. 5 shows that the average performance of the deep learning system is similar to the expert performance for both the left and right canals, but the deep learning system can be seen to perform better than the experts near the mandibular foramen for the right canals. It can also be seen that there are no major differences in the deep learning system and the radiologists’ performances between the left and the right canals. Visual illustration of the average performance is presented in Fig. 5b for the left canal and in Fig. 5c for the right canal.

### Qualitative assessment

**Figure 6:**
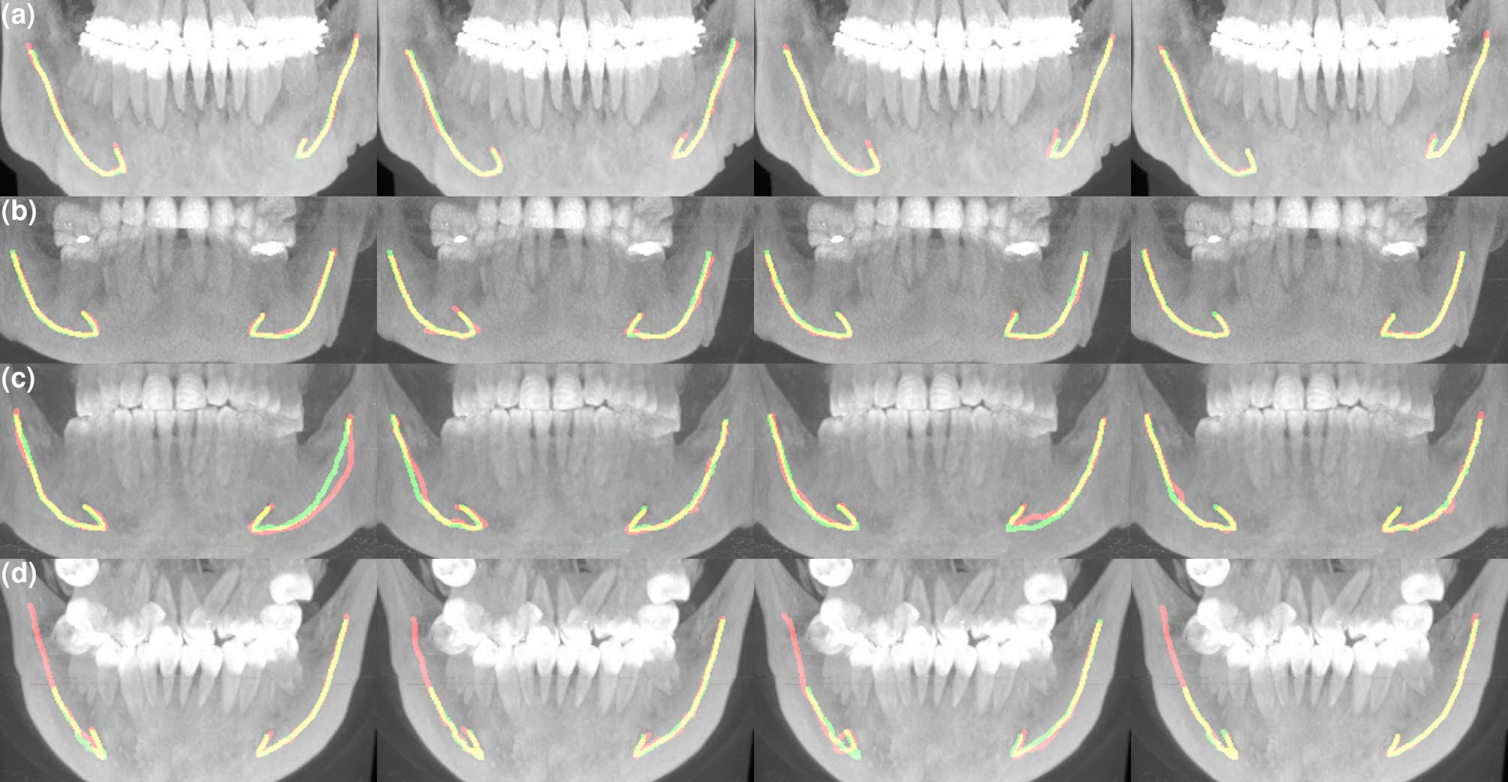
Maximum intensity projection of CBCT with overlaid expert and DLS segmentations. Every image on each column is annotated by the same expert, shown in red, DLS annotation shown in green, and overlap shown in yellow. (**a**) GiANO HR with 0.3 mm spacing, (**b**) a DentiScan with 0.4 mm spacing, and (**c**,**d**) DentiScan with 0.25 mm spacing, (**a**,**b**) Low interobserver variability (IV) and low DLS to expert variability (DV) on a scan with no heterogeneities and the both canals were labelled as Clear. (**c**) High IV with a scan labelled with difficult bone structure (DBS) and the left canal was labelled Unclear. (**d**) High DV where the DLS outcome was incomplete with a scan labelled with DBS, movement artefact, and the right canal labelled Unclear.

The qualitative analysis of the test images was done visually by a senior radiologist, who compared the deep learning outcome and the annotations of each radiologist. The deep learning system produced three significant errors (> 1/3 of the canal was missing) out of 300 evaluated with one segmentation error and two post-processing errors. The likely cause for the segmentation error was a technical artefact of the CBCT machine. Other 297 out of 300 (99%) canals analysed were correct. Figure 6 presents four example scans from the test dataset using maximum intensity projection, each overlaid with the segmentation from the DLS and the Experts.

The qualitative interobserver variance and the variance between the deep learning system and radiologists can be classified into two categories, i.e. differences in markings and human errors. The differences in markings were caused by the preference of each radiologist in selecting the middle of the canal and the spacing between markings. This in turn is due to differences in human anatomy such as difficult bone structure, bone porosity, pathological conditions, variable thickness, shape and curvature areas of the canal path, and other sources like metallic structures and imaging artefacts, thus making standardised markings difficult or even impossible. However, this variance turned out not to be substantial. In contrast, the variance due to human error was found to be considerable, i.e. 29 out of 1200 (i.e. 2.4%) human annotations were outside the actual canal for a few markings, but appeared in the areas not considered to have major clinical relevance. There were no major differences in error rate between the Experts.

## Discussion

To summarise, the deep learning system can accurately segment the mandibular canal with better performance than the radiologists for a clinically and technically heterogeneous dataset with statistical significance. In addition, we have demonstrated that the previously introduced system generalises to new devices and to a geographically and ethnically separate clinical data with statistically significant differences with a lower median SMCD on DentiScan and GiANO HR, and with a higher median SMCD on Viso G7.

In the pairwise comparison, when measured with the median of the mean curve distance and with the proportion of canal being within 2 mm radius, the DLS had a similar performance to the pairwise comparison between the Experts. Specifically, there was better agreement between the DLS and Expert 3 and Expert 4, than with Expert 1 and Expert 2, which may be due to their role in the development set annotation. We note that the highest agreement was with Expert 4 even though most of the development set was annotated by Expert 3. When compared with the highest disagreement, the DLS showed lower median disagreement than the interobserver disagreement with a statistically significant difference. In the device-wise comparison, there were statistically significant differences between the DLS performance and interobserver variability with lower disagreement between the DLS and radiologists for the ProMax 3D Mid, DentiScan, and GiANO HR, but not for the Scanora3Dx and Viso G7 scanners. When the Experts and the DLS were compared to a majority vote consensus segmentation, the DLS gave the lowest median of symmetric mean curve distance with statistically significant difference to the Experts. In addition, the deep learning system turned out to have similar variability in the anatomic localization specific error as the human observers had, with the largest errors appearing near the mandibular foramina.

The generalisation capability of the artificial intelligence algorithms in radiology is considered to be one of challenges, due to large variances between the imaging parameters, such as protocols, technical solutions, field of view, imaging parameters, and voxel sizes, as well as heterogeneities such as patient anatomy and pathology^2^. In addition, the CBCT image quality is affected by patient movement and metallic artefacts caused by dental or oral surgical materials^17–19^. Despite this, we observed that the deep learning system had similar performance across the different imaging devices as well as a variety of patient specific heterogeneities. When measuring the out-of-distribution generalisation using the previously published system, which was trained with scans from the ProMax 3D Mid and Scanora 3Dx from a single hospital, the performance appeared to be practically similar across all evaluated devices and both imaging centres for the majority of cases. However, definite conclusions about the deep learning system or interobserver performance in case of ethnic variability are left for future work with a larger dataset and controlled study protocol.

The visual quality assessment of the DLS segmentation revealed negligible amounts of errors, which were dissimilar to the errors produced by the radiologists. Notable errors produced by the DLS were incomplete canals, whereas the radiologists’ errors were mostly deviations of the canal path, in both cases re-annotation would be required. The qualitatively assessed error rate was 1% for the DLS and 2.4% for expert annotators, which demonstrates at least comparable performance to human professionals.

These results indicate that the deep learning system could be utilised in maxillofacial radiology for automatic segmentation of the mandibular canal to augment expert annotators. The applicability of deep learning for mandibular canal segmentation from volumetric imaging data is promising and encourages continuing to limited clinical testing and validation under surveillance of radiologists. After clinical validation and acceptance to clinical use, DLS can be considered as a valuable tool in everyday dental, surgical and radiological work to avoid complications in minor and major operations, and reduce time in computer assisted operation planning and authoring radiological reports. Most common operations are mandibular third molar extractions and implantology. The third mandibular molar or its multiple roots may be in close proximity with the mandibular canal. In addition, the DLS may improve visualisation of anatomical relations, in comparison to stationary reconstruction images, and reading radiological reports, as well as reduce the time consumed in implantological or other surgical planning. Moreover, reporting the anatomical relations of the mandibular canal to other anatomical or pathological structures is of paramount importance in radiological work, where DLS assisted visualisation would be time- and labour saving. In addition, these outcomes could encourage deep learning approaches for other clinical and research tasks with CBCT and computed tomography scans such as tissue segmentations, cephalometric landmark detection, bone density estimation, and utilisation of ALARA (as low as reasonably achievable) principle to minimise harmful effects of the ionising radiation.

### Limitations

We acknowledge the following limitation of the deep learning system: deep learning neural networks that are trained without defining diagnostically important features may have an inherent limitation of possibly learning features that are unknown or ignored by medical experts. In addition, the evaluation dataset did not include children.

## Conclusions

Within the limitations of our study the following conclusion can be drawn. The DLS showed lower variability than the interobserver variability of the radiologists. The out-of-distribution generalisation capability of the DLS to new CBCT scanners and ethnic groups of patients not used in model training was found to be similar across all evaluated devices and both radiological centres for the majority of cases, showing promising temporal and geographic generalisability. When compared to radiologists’ consensus segmentation as a *gold standard*, the DLS had a lower symmetric mean curve distance than the radiologists. These results encourage integration of the DLS into clinical workflow under control of radiologists.

## Supplementary Information

Supplementary Information.

## Data Availability

The datasets used in model training, validation, and testing were provided by TAUH and CMU, and as such is not publicly available and restrictions apply to their use according to the Finnish law and General Data Protection Regulation (EU) and to the Thai law, respectively.

## Code availability

The code used for pre- and postprocessing and the deep learning techniques includes proprietary parts and cannot be released publicly. However, the proposed method can be replicated using the information in the “Methods” section.
