# Supplementary material for: Comparison of deep learning segmentation and multigrader-annotated mandibular canals of multicenter CBCT scans
Source: Sci Rep. 2022 Nov 3;12:18598. doi: 10.1038/s41598-022-20605-w (PMC9633839; doi:10.1038/s41598-022-20605-w)
Supplement: Supplementary file 1 — Supplementary Information. [file 41598_2022_20605_MOESM1_ESM.docx]

Supplementary Information

## Supplementary results

The development set was divided into the training and internal validation sets with the latter being randomly selected to have uniform distribution of the five CBCT scanners. The full distribution of scans and patients for all of the subsets are shown in Supplementary Table S1.

**Supplementary Table S1**: **Distribution of scans and patients for training, internal validation, and holdout test subsets.**

| **Manufacturer and device** | **Development** | | | | **Holdout Test set** | |
| --- | --- | --- | --- | --- | --- | --- |
|  | **Train set** | | **Internal Validation set** | |  |  |
|  | Scans | Patients | Scans | Patients | Scans | Patients |
| Planmeca ProMax (3D, 3D Max, 3D Mid) | 599  (68 %) | 570  (67 %) | 20  (20 %) | 20  (20 %) | 30  (20 %) | 30  (20 %) |
| Planmeca Viso G7 | 75  (9 %) | 75  (9 %) | 20  (20 %) | 20  (20 %) | 30  (20 %) | 30  (20 %) |
| Soredex Scanora 3Dx | 74  (8 %) | 74  (9 %) | 20  (20 %) | 20  (20 %) | 30  (20 %) | 30  (20 %) |
| NSTDA DentiScan | 70  (8 %) | 70  (8 %) | 20  (20 %) | 20  (20 %) | 30  (20 %) | 30  (20 %) |
| NewTom GiANO HR | 64  (7 %) | 64  (8 %) | 20  (20 %) | 20  (20 %) | 30  (20 %) | 30  (20 %) |
| **Total** | **882** | **853** | **100** | **100** | **150** | **150** |

**Supplementary Table S2**: **Comparison of performances with respect to the reference ground truth**^a^**.**

| **Assessment** | **Median**  **of SMCD (mm)** | **IQR**  **of SMCD (mm)** | **Mean**  **of SMCD (mm)** | **Standard deviation of SMCD (mm)** |
| --- | --- | --- | --- | --- |
| Expert 1 | 0.624 | 0.230 | 0.682 | 0.379 |
| Expert 2 | 0.550 | 0.219 | 0.621 | 0.392 |
| Expert 3 | 0.469 | 0.136 | 0.518 | 0.376 |
| Expert 4 | 0.423 | 0.143 | 0.467 | 0.396 |
| DLS | 0.392 | 0.113 | 0.460 | 0.390 |

^a^All the Experts and the model perform differently with statistical significance computed using all pairs of Wilcoxon signed rank tests (*p* < 0.001).

Disagreement measured with interobserver variability and the deep learning system to Expert variability both increase when a heterogeneity is present or the canal is subjectively marked as Unclear. We show that there is statistically significant difference in IV and DV variability for scans with any vs. no heterogeneities. However, there are typically multiple heterogeneities present in a single scan, thus making conclusions difficult. Full comparisons for each of the heterogeneities are shown in Supplementary Figure S1.

| 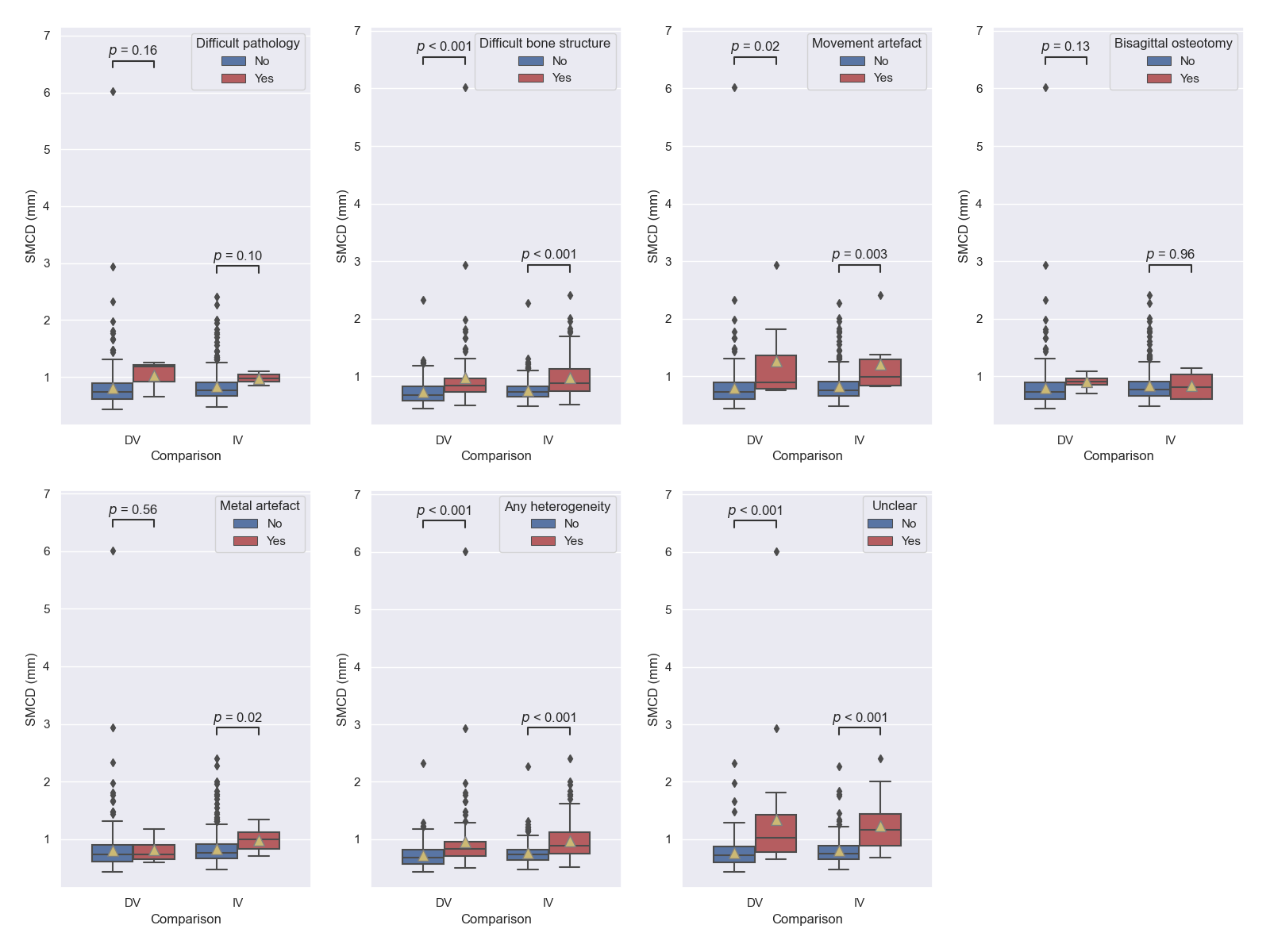 |
| --- |
| **Supplementary Figure S1**: **Boxplot comparison of disargemeent measured with interobserver variability (IV) and the deep learning system to expert variability (DV) grouped by heterogeneities.** Results are measured with the symmetric mean curve distance (SMCD) in mm. Statistically significant differences with alpha value of 0.001, measured by two-tailed Mann-Whitney U-test, between the unpaired subgroups, are seen due to difficult bone structure, movement artefact, any heterogeneity, and Unclear for both IV and DV. In addition, a statistically significant difference is seen due to metal artefacts on the IV but not on DV. |

| 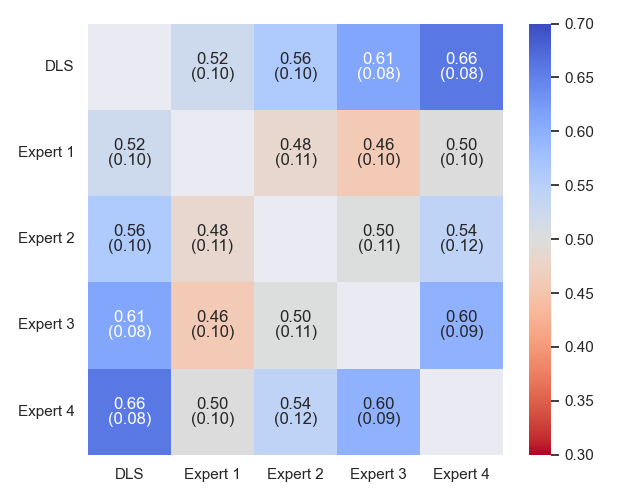 |
| --- |
| **Supplementary Figure S2**: **Pairwise comparison of the experts and the deep learning system (DLS) measured with Dice similarity coefficient (DSC).** Each row represents which assessment was used as the ground truth and each column which was used as the estimate. Reporting mean value with standard deviation in parenthesis. |

| 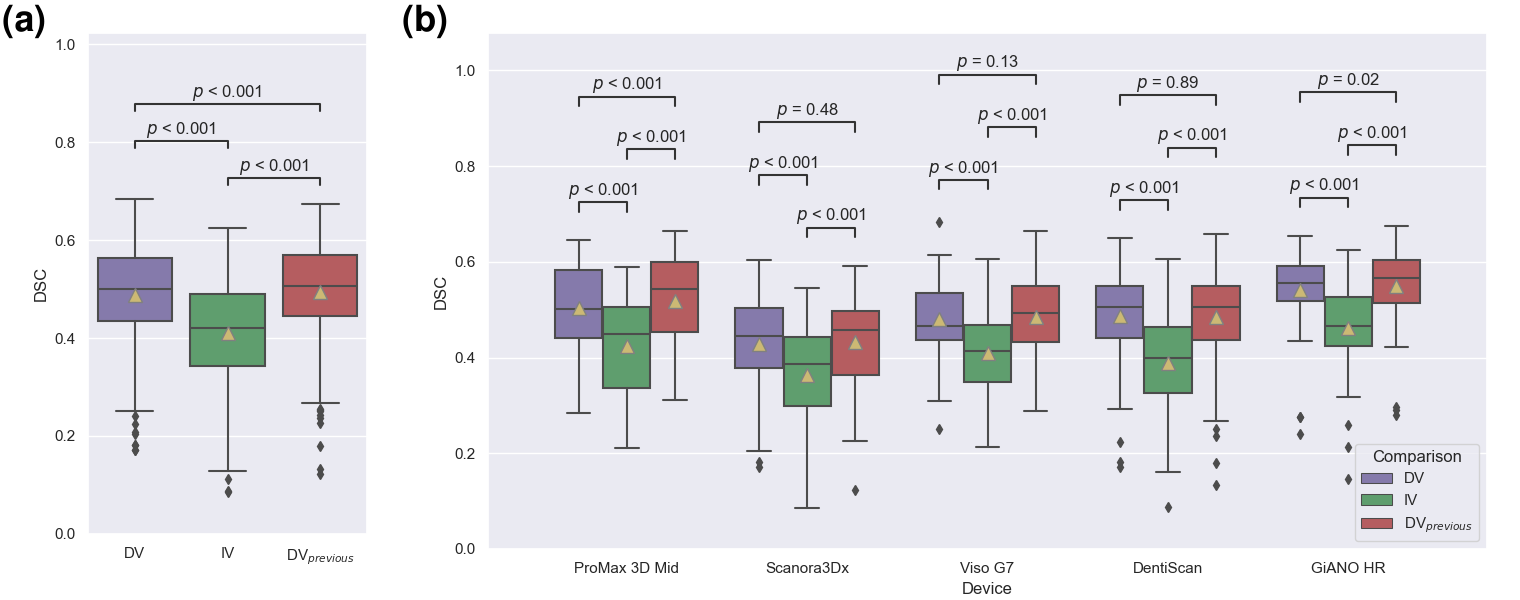 |
| --- |
| **Supplementary Figure S3**: **Tukey’s boxplot comparison of interobserver variability (IV), DLS to expert variability (DV), and previous method^4^ to expert variability (DV_previous_), measured with Dice similarity coefficient (DSC)**. Statistical significance measured with two-tailed Wilcoxon signed-rank test. (**a**) Comparison of full test dataset (N=300). (**b**) Device-wise comparison between the groups (N=60 per device). |

| 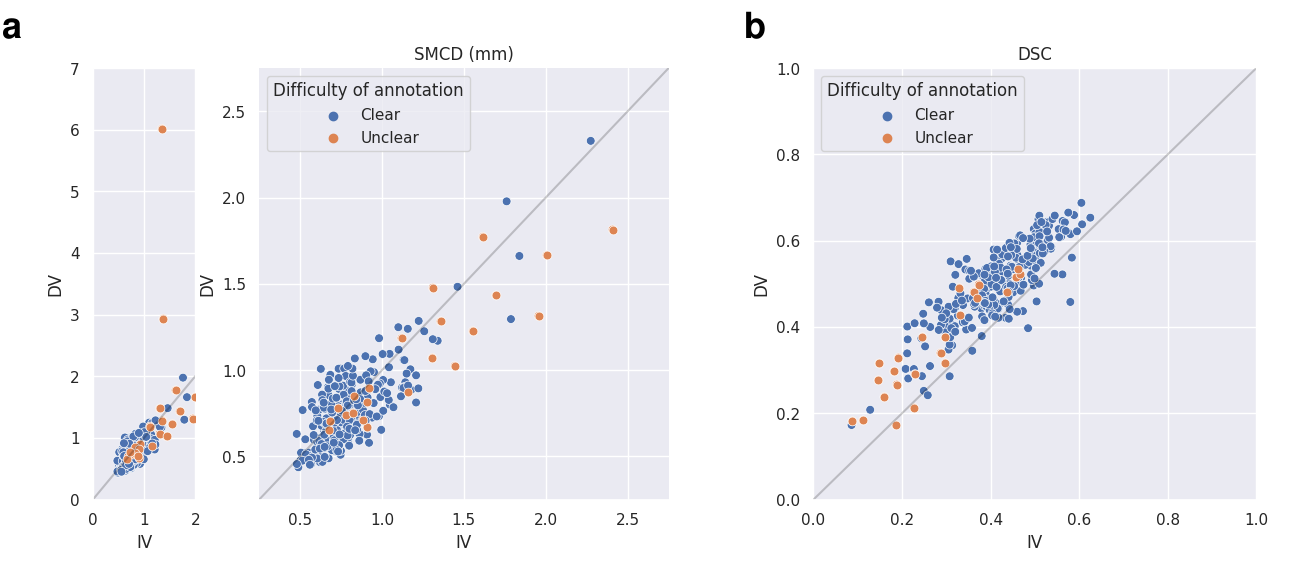 |
| --- |
| **Supplementary Figure S4**: Scatterplot comparison between interobserver variability (IV) and the deep learning system to expert variability (DV) with Clear and Unclear canals colored in blue and orange, respectively. (**a**) Comparison measured with symmetric mean curve distance (SMCD) in mm. The left subfigure includes the two outlier cases and the right subfigure includes a closer view excluding the outliers. (**b**) Comparison measured with Dice similarity coefficient (DSC). |

| 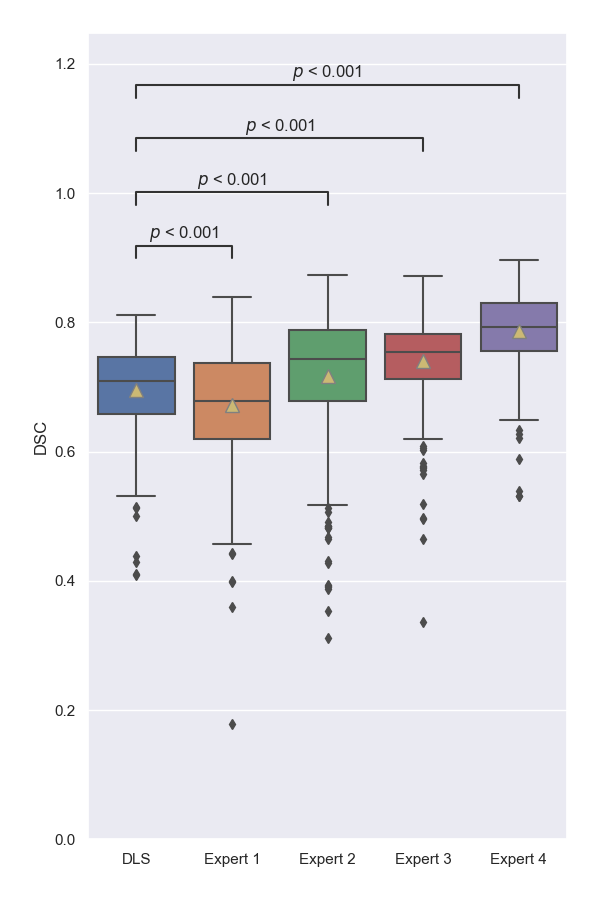 |
| --- |
| **Supplementary Figure S5: Tukey’s boxplot comparison of the performances of DLS and the experts against the reference segmentation measured in Dice similarity coefficient (DSC).** Statistical significance measured with two-tailed Wilcoxon signed-rank test. |

## Symmetric Mean Curve Distance

The symmetric mean curve distance (SMCD) between two curves is calculated as an average of the mean curve distances (MCDs) computed both ways between them. In other words, one of the curves is first set as the *ground truth* and the other as the *estimator* of that curve, and the MCD is calculated between them. Then, the roles are switched and the MCD is calculated again. The result is the sum of the two MCD values divided by two. Supplementary Figure S6 visualises a synthetic pair of two dimensional curves and the two possible ways to compute the MCD. We can see that the curves have differences from translation, but also the ends of the curves are different. This causes the MCD values to differ depending on which of the curves is assigned to which of the roles. However, the SMCD takes both of the MCD values into account and thus it is affected by disagreements on both ends of the canals. In the main experiments, the curves are in three dimensions and the distances are calculated accordingly.

| 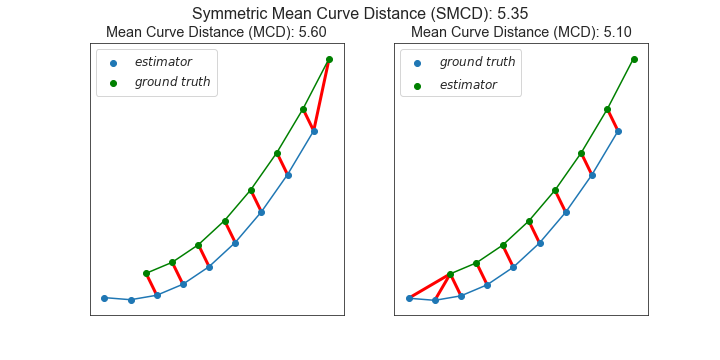 |
| --- |
| **Supplementary Figure S6: A visual illustration of curve distances on two synthetic curves.** Here a green curve and a blue curve are compared against each other. The curves are represented by a collection of coordinates, which are visualised as dots. The red lines represent the distance of the closest point on the *estimator* curve to each point on the *ground truth* curve. The MCD values are calculated as the average of these distances, whereas the SMCD is the average of the two MCD values. The synthetic curves are two dimensional for visualisation purposes. |
